# Supplementary material for: Intermittent preventive treatment with sulphadoxine-pyrimethamine but not dihydroartemisinin-piperaquine modulates the relationship between inflammatory markers and adverse pregnancy outcomes in Malawi
Source: PLOS Glob Public Health. 2024 May 16;4(5):e0003198. doi: 10.1371/journal.pgph.0003198 (PMC11098340; doi:10.1371/journal.pgph.0003198)
Supplement: Attachment — Submitted filename: Cheng et al Response to Reviewers.docx [file pgph.0003198.s003.docx]

Dear Alassane Dicko and Reviewers,

Thank you for your comments on our submission ‘Intermittent preventive treatment with sulphadoxine-pyrimethamine but not dihydroartemisinin-piperaquine modulates the relationship between inflammatory markers and adverse pregnancy outcomes in Malawi’ by Kaylene Cheng and others. Please see below how we have addressed your queries.

Regards,

Elizabeth Aitken and co-authors

**Journal Requirements:**

1. Please review your reference list to ensure that it is complete and correct. If you have cited papers that have been retracted, please include the rationale for doing so in the manuscript text, or remove these references and replace them with relevant current references. Any changes to the reference list should be mentioned in the rebuttal letter that accompanies your revised manuscript. If you need to cite a retracted article, indicate the article’s retracted status in the References list and also include a citation and full reference for the retraction notice.

We have reviewed our reference list.

2. Please include a complete copy of PLOS’ questionnaire on inclusivity in global research in your revised manuscript. Our policy for research in this area aims to improve transparency in the reporting of research performed outside of researchers’ own country or community. The policy applies to researchers who have travelled to a different country to conduct research, research with Indigenous populations or their lands, and research on cultural artefacts. The questionnaire can also be requested at the journal’s discretion for any other submissions, even if these conditions are not met.  Please find more information on the policy and a link to download a blank copy of the questionnaire here: https://journals.plos.org/globalpublichealth/s/best-practices-in-research-reporting. Please upload a completed version of your questionnaire as Supporting Information when you resubmit your manuscript.

We have completed the PLoS questionnaire on inclusivity in global research and have included it in the submission.

3. Please provide separate figure files in .tif or .eps format only and remove any figures embedded in your manuscript file. Please also ensure all files are under our size limit of 10MB.

For more information about figure files please see our guidelines:

https://journals.plos.org/globalpublichealth/s/figures 
<https://journals.plos.org/globalpublichealth/s/figures#loc-file-requirement>

The figure is now submitted as a TIF.

3. We do not publish any copyright or trademark symbols that usually accompany proprietary names, eg  ©, ®, ™  (e.g. next to drug or reagent names). Please remove all instances of trademark/copyright symbols throughout the text, including ® & ™ on page 6.

We have removed these symbols from the manuscript.

5. In the online submission form, you indicated that "The data that support the findings of this study are available from the corresponding author upon reasonable request". All PLOS journals now require all data underlying the findings described in their manuscript to be freely available to other researchers, either 1. In a public repository, 2. Within the manuscript itself, or 3. Uploaded as supplementary information.
This policy applies to all data except where public deposition would breach compliance with the protocol approved by your research ethics board. If your data cannot be made publicly available for ethical or legal reasons (e.g., public availability would compromise patient privacy), please explain your reasons by return email and your exemption request will be escalated to the editor for approval. Your exemption request will be handled independently and will not hold up the peer review process, but will need to be resolved should your manuscript be accepted for publication. One of the Editorial team will then be in touch if there are any issues.

We have written to the editorial team regarding availability of data, in brief it is to be uploaded to WWARN but this is yet to happen, the editorial team has said it is possible to finalise the data availability statement until after completion of peer review.

See email copied below.

Dear Dr Aitken,

Thanks for reaching out to us with your query, I'm happy to provide some assistance in this matter.

We can delay finalizing the manuscript's Data Availability statement until after completion of peer review should your manuscript be accepted for publication. If you believe that you would have the data upload to the WWARN database sorted out by that point then we should be okay to proceed for now to if you note this in your resubmitted Data Availability statement. We would just need for the data to be uploaded and available before proceeding to publication. 

Uploading the the raw data for the inflammatory markers as supporting information files should be perfectly acceptable.

Please let me know if you have any additional questions or concerns, I'm here to help!

Kind regards,

Adam Thompson
Associate Peer Review Operations Specialist

PLOS ONE | plosone@plos.org
Empowering researchers to transform science
1265 Battery Street, Suite 200 San Francisco, CA 94111

Case Number: 08426979
ref:!00DU00Ifis.!500PM09BRnh:ref

**Comments to the Author**

Please use the space provided to explain your answers to the questions above. You may also include additional comments for the author, including concerns about dual publication, research ethics, or publication ethics. (Please upload your review as an attachment if it exceeds 20,000 characters)

**Reviewer #1:** In this manuscript, the authors aimed to investigate if SP alters the relationship between inflammation and birth outcomes. The study describes that elevated AGP levels at delivery were associated with adverse birth outcomes in women that received DP or DP+AZ and concluded that SP modifies the relationship between inflammation and adverse outcomes.
One of my major comments is that the proportions of women with elevated AGP and CRP were similar between groups (Table 2), therefore, how does SP modify the relationships between inflammatory markers and outcomes if proportion of women with elevated levels are similar? For SP to change/reduce the risks, wouldn’t that be associated or even required to have lower levels of inflammatory markers?

Yes, the number of individuals with inflammation is similar in all three groups. The data presented in this paper suggests that the changed relationship between inflammation and outcome isn’t due to simply one of the groups having less inflammation than another group but something else. It is possible that there are for example different causes for inflammation in the three groups and this is what is causing the different patterns, or possibly SP receipt counteracts the ability for inflammation to cause adverse outcomes.

The authors should also show if CRP and AGP levels (as continuous variables) are similar between groups at the 3 time points.

We have included this analysis in figure 1 (G&H), levels (continuous variables) were similar between groups at all the 3 time points.

Line XXX “Levels of CRP and AGP and proportions of women with elevated AGP or CRP were similar in each of the three treatment arms at all three timepoints (Figure 1 & Table 2).”

**Comments**
1. Abstract, lines 51-52: without looking at the tables, this sentence implies that there are no differences between the groups. However, according to Table 4, elevated AGP levels at enrollment in the DP+AZ group was associated with increased SGA, and in the DP group, elevated AGP did not increased the risk of any adverse outcome.

We have edited the abstract to note the association of SGA in the DP+AZ group. See lines copied below.

Line 51 “Elevated AGP at enrollment was associated with adverse birth outcome (aRR 1.40, 95% CI: 1.15, 1.70), with similar associations observed across treatment arms, exceptions being that elevated AGP was associated with low maternal weight gain in SP recipients (aRR 1.94, 95% CI: 1.36, 2.76) and with SGA in DP+AZ recipients (aRR 1.49, 95% CI 1.02, 2.17).”

2. Table 1: Are there any significant differences in birth outcomes (proportions) between the groups?

The proportions of individuals with adverse birth outcomes in the groups is published in table 1. The analysis of the IMPROVE trial (in which approximately three times as many women participated as in this substudy) and the impacts of IPTp with SP, DP or DP+AZ has been published by Madanitsa et al 2023 Lancet, which is referenced throughout the paper. Whether there are significant differences in pregnancy outcomes between arms in our subset of participants is not relevant to our analysis

3. Infection status: what was the infection rate at the 3rd visit? At delivery, do the authors have placental blood smear or PCR results that can be included in the analysis.

We do have infection data for visit 3 microscopy and (in a small subset; note missing data) by PCR. We also have delivery placental microscopy data. We have added these data to table 1.

4. How do you explain the reduction in AGP and CRP at visit 3 that was close to delivery in some, followed by increased levels at delivery?

We suspect that some of the decrease from enrolment to visit 3 is likely due to the anti-inflammatory effects of effective prevention of malaria and possible effects against other infections, as the decrease was more pronounced in the DP+AZ group compared to the SP group; however other factors likely also contribute as similar decreases in these inflammatory markers have also been previously seen in other cohorts who did not receive IPTp (see paragraph Line 382 discussion).

The increase in CRP (and possibly AGP) at delivery could be due to the inflammatory nature of labour. The use of samples taken at delivery is noted as a weakness in the study (line 418).

5. Table 4, enrollment: because samples at enrollment were collected prior to drug administration, why AGP levels are associated with SGA or any adverse effect only in the DP+AZ group? How do you explain differences at enrollment based on treatment groups?

Even though the inflammation was detected at enrolment, the outcome was still assessed at delivery (3-6 months after first receipt of the drugs) providing time for the antimalarials to modify the association between the markers of inflammation and outcome.

6. Similarly in Table 5, AGP levels at enrollment were associated with reduction in birthweight and gestational age in SP and DP+AZ groups but not in DP, and lower maternal weight gain in the SP group only, why is that? CRP levels at enrollment were associated with a reduction in birthweight only in the DP+AZ group but not in other treatment groups, please explain.

Regarding AGP at enrolment and associations with reduced birthweight, though it is not significant in the DP group, there was weak evidence of difference in this group (P=0.09) and the direction and size of association was similar to what was seen in both the SP and DP+AZ groups. In contrast, the association between maternal weight and elevated AGP at enrolment was completely absent from the DP+AZ group (P=0.35), while there was very strong evidence it was present in the SP group (P=0.001), why this association was seen so strongly in women receiving SP but not in those receiving DP +AZ may be linked to the fact that SP has previously been associated with better maternal weight gain (see paragraph line 401).

The lack of association with gestational age in the DP group has now been highlighted in the results section of the manuscript.

Line 280“There was no association with AGP and gestation length in the DP arm (Table 5)”.

7. There is no reference in the paper to the increased gestational age associated with CRP at visit 3 in the DP+AZ group. The authors should comment on that.

There is no increase in gestational age at with CRP at visit 3 but there is one at delivery. We previously mentioned this when describing the combined DP & DP+AZ results but have now specified that the relationship is in the DP+AZ group (see line 301).

8. Lines 288-294: elevated AGP levels at delivery were associated with increased LBW, PTB, foetal loss, reduced birthweight and gestational age after combining the DP with the DP+AZ group. This is not clear in the text. Please clarify that these associations were not observed in each of the groups alone.

We have clarified the results in the text.

Line 293: “At delivery, elevated AGP was not associated with adverse birth outcomes in women receiving SP (Table 4). By contrast, in women receiving either DP or DP+AZ, elevated AGP at delivery was associated with increased risks of LBW (aRR 1.48, 95% CI: 1.01, 2.18), PTB (aRR 2.37, 95% CI: 1.44, 3.90), foetal loss (aRR 3.83, 95% CI: 1.18, 12.38) and evidence of an increased risk for SGA (aRR 1.30, CI 0.99, 1.71), however within the individual DP and DP+AZ groups elevated AGP at delivery was not significantly associated with foetal loss or low birthweight. Women receiving either DP or DP+AZ with elevated AGP had babies 70 g smaller than those without elevated AGP (-95% CI: -140, -0.2)(Table 5) and elevated CRP at delivery was associated with a decreased risk of foetal loss in women receiving either DP or DP+AZ (aRR 0.31, 95% CI: 0.10, 0.90) (Table 4) (note neither of these associations were significant in the individual DP or DP+AZ groups). Elevated CRP at delivery was also associated with a longer gestation in women receiving DP+AZ (4.99 days, 95% CI: 1.73, 8.25)(Table 5).”

9. Lines 347-348: “Associations between inflammation and adverse outcomes were seen in all three arms at enrolment”, this isn’t the case in the DP group (Tables 4 and 5)

It is true that for the DP arm there were not P values less than 0.05 at enrollment, however there was weak evidence of difference between elevated AGP and any adverse outcome in the DP group (P=0.08) and for reduced birthweight (P=0.09) and importantly the direction and size of the associations for the DP arm at enrolment were similar to those of the SP and DP+AZ groups (Table 4) (Table 5), and so we have concluded that associations between inflammation and adverse outcomes were seen in all three arms. The specific P values and co-efficient for each association are included in the manuscript so the strength of this data is clear to the reader.

10. Line 348-349: for clarity, the authors should change to “restricted to the DP+AZ and DP arms combined”.

Associations between adverse outcomes and inflammation were see in both the DP and DP+AZ groups as well as the combined DP and DP+AZ group, however some associations were only statistically significant when the two groups were combined.

We have edited the text to make it clearer

Line 350 “these associations were restricted to the DP+AZ and DP arms (note, some associations reached significance only in the combined DP & DP+AZ group), suggesting that SP may dampen the relationship between inflammation at delivery and adverse birth outcomes.”

Similarly, clarify in the Abstract (line 54) that the results are for the 2 groups combined.

Thanks for the suggestion, we have edited the text.

Line 55 “At delivery, women with elevated AGP receiving either DP or DP+AZ had an increased risk of adverse birth outcomes (aRR 1.60, 95% CI: 1.28, 2.00), including LBW, PTB and foetal loss. This association was not seen in women receiving SP (aRR 0.82, 95% CI: 0.54, 1.26).”

11. Lines 361-367: in the current study AGP at enrollment was associated with a composite of adverse outcomes, and in PNG at delivery. Is it correct that in the current study, AGP levels at delivery (all women) were not associated with adverse outcomes?

We did not look at associations between inflammatory markers and adverse outcome at delivery in all women together as we felt that the modulatory effects of the antimalarials was so great that we should not pool the three treatment arms for timepoints after enrolment.

At what time point AGP levels measured in the Tanzanian study?

Thank you for pointing out that the time point for the Tanzanian study by Kirby et al was not included, we have amended this.

Line 365 “in Tanzania increased AGP at 32 weeks’ gestation was associated with PTB and lower birth weight.”

12. Lines 412-414: Previous studies described increased levels of acute phase proteins at delivery in women that had spontaneous vaginal delivery vs C-section, the models can be adjusted for that if applicable here.

We agree with the reviewer that adjusting for vaginal delivery or C-section may improve the models however we do not have the data to do this. Also, as the population of Malawian women who have a c-section in general is low (less than 10%, Betran et al 2016 PLoS One) it is possible that this extra information may not greatly affect the models.

13. Did the authors examined the effect of malaria infection on the association between inflammatory markers and birth outcomes, i.e., including malaria infection as a covariate in the models.

In the original analysis we did not include malaria infection as a co-variate in the model.

To address the reviewer’s query we have since assessed the effect of malaria by performing an adjusted analysis (including whether women had any Plasmodium spp. infection during pregnancy as a covariate in the model) and found that including malaria infection did not change the relationship between inflammatory markers and risk of adverse foetal outcome in any of the arms at any timepoint. We have included this analysis in this letter however as the results are almost identical to what the original analysis showed we have not included it in the revised manuscript.

###### Elevated CRP and AGP levels during pregnancy and risks of any adverse foetal outcome, by treatment arm, including malaria in model

| **SP** | | **Any adverse foetal outcome** | |
| --- | --- | --- | --- |
|  |  | **aRR (95% CI)** | **P** |
| **Enrolment (Week 16-28)** | **CRP** | 1.23 (0.87, 1.72) | 0.24 |
|  | **AGP** | 1.40 (0.99, 1.98) | 0.059 |
| **Visit 3** | **CRP** | **1.50 (1.00, 2.23)** | **0.048** |
|  | **AGP** | 1.49 (0.90, 2.46) | 0.12 |
| **Delivery** | **CRP** | 0.85 (0.57, 1.27) | 0.43 |
|  | **AGP** | 0.82 (0.53, 1.26) | 0.36 |
| **DP** | |  |  |
| **Enrolment (Week 16-28)** | **CRP** | 1.07 (0.79, 1.45) | 0.64 |
|  | **AGP** | 1.31 (0.97, 1.77) | 0.08 |
| **Visit 3** | **CRP** | 1.43 (0.98, 2.08) | 0.059 |
|  | **AGP** | 1.19 (0.71, 1.97) | 0.51 |
| **Delivery** | **CRP** | 0.95 (0.66, 1.36) | 0.77 |
|  | **AGP** | **1.63 (1.20, 2.22)** | **0.002** |
| **DP+AZ** | |  |  |
| **Enrolment (Week 16-28)** | **CRP** | 1.34 (0.99, 1.82) | 0.058 |
|  | **AGP** | 1.49 (1.09, 2.03) | 0.013 |
| **Visit 3** | **CRP** | 1.22 (0.79, 1.89) | 0.36 |
|  | **AGP** | 1.41 (0.87, 2.29) | 0.16 |
| **Delivery** | **CRP** | 0.94 (0.66, 1.33) | 0.73 |
|  | **AGP** | **1.56 (1.14, 2.15)** | **0.006** |
| **DP & DP+AZ** | |  |  |
| **Enrolment (Week 16-28)** | **CRP** | 1.20 (0.97, 1.49) | 0.10 |
|  | **AGP** | 1.39 (1.11, 1.74) | 0.004 |
| **Visit 3** | **CRP** | 1.33 (1.00, 1.77) | 0.052 |
|  | **AGP** | 1.30 (0.91, 1.84) | 0.15 |
| **Delivery** | **CRP** | 0.94 (0.73, 1.22) | 0.65 |
|  | **AGP** | **1.59 (1.28, 2.00)** | **<0.0001** |

The composite adverse birth outcome variable includes: Low birth weight (LBW) defined as < 2500 g, preterm birth (PTB), defined as <37 gestational weeks, foetal loss defined as a non-live birth, small for gestational age (SGA) defined as birth weight lower than the 10^th^ centile of the intergrowth-21^st^ standards (1) and/or neonatal loss Adjusted Risk Ratios (aRR) and 95% confidence intervals were estimated using logistic regression and adjusted for maternal age, maternal height, mid-upper arm circumference, gravidity, number of antenatal visits, study site, malaria infection, and included an interaction term between inflammatory marker and treatment arm. Significance is defined as P <0.05 and significant findings are highlighted with bold text.

Minor comments
1. Line 97: “a subset of IMPROVE cohort”, please describe how the subset was selected.

For the specified time points, we used all available plasma samples from the Malawi arm of the study. The below has been added to the methods.

Line 118 “All available plasma samples for the three selected timepoints from Malawi were used.”

2. A new article presenting a model study to explain the mechanism associated with increased birthweight in women that received IPTp-SP was recently published (https://doi.org/10. 1016/j.ebiom.2023. 104921). The authors should consider referencing the study.

Thank you for highlighting this paper (Kim et al eBioMedicine December 2023), we have included it in the manuscript.

Line 394 “Recent evidence using in-vitro models suggests that SP may directly improve the ability of the intestine to absorb nutrients in a nutrient deficient environment and suppresses the inflammatory responses associated with nutrient deficiency (2).

Reviewer #2: The authors address the modulation of the inflammation by the antimalarial compounds given as intermittent preventive treatment. This is a very important topic as there is strong evidence that SP-IPTp has significantly contributed to the reduction of low birth weight and other adverse pregnancy outcomes. With the occurrence of resistance to SP, alternatives are being evaluated, but recent studies have still shown that although SP did not have a strong antimalarial effect as mefloquine or dihydroartemisinin-piperaquine, it still showed good effect in preventing low birth weight, suggesting there could be another mechanism beyond the prevention of malaria.
The authors have assessed the CRP and AGP as inflammatory markers and they have well analysed and described their findings and also pointed out the limitations of their analysis and suggested further studies to continue to explore the question. The findings are worth being published.
The following minor comments need to be addressed:
Be consistent with the date format. See line 110 and line 117.

We have gone through and made date formats consistent
Line 209: correct anti-natal

Thank you- we have corrected this to ante-natal

Line210: correct Kenua

Thank you- we have corrected this to ‘Kenya’.

From line 220: consider providing also absolute values within brackets so that the reader can check. For example instead of 35.7%... only, consider 35.7% (xx/xx)...

We have modified the text as suggested to include the numerator and denominator.

Line 218 “CRP concentrations were elevated in 35.7% (475/1332), 16.4% (181/1102) and 74.8% (818/1094) of women at enrolment, visit 3 and delivery, respectively. Regarding AGP, 31.3% (417/1332), 9.4% (104/1102) and 32.5% (356/1094) of women had AGP concentrations in the upper quartile at enrolment, visit 3 and delivery, respectively (Table 2).”

Reviewer #3: This manuscript reports result of a study on “Intermittent preventive treatment with sulphadoxine-pyrimethamine but not dihydroartemisinin-piperaquine modulates the relationship between inflammatory markers and adverse pregnancy outcomes in Malawi”, by Kaylene et al.

They found that Elevated alpha-1-acid glycoprotein (AGP)AGP at enrollment was associated with adverse birth outcome, with similar associations observed across treatment arms, and
with low maternal weight gain in SP recipients only. At visit 3 there were few associations between inflammation and adverse outcomes. At delivery, women with elevated AGP receiving DP or DP+AZ had an increased risk of adverse birth outcomes, including LBW, PTB and foetal loss. The risk of an association between elevated AGP and adverse birth outcome was higher in those receiving DP or DP+AZ compared to those receiving SP. In contrast to AGP, no clear associations between CRP and adverse outcomes were observed. The authors concluded that SP modifies the relationship between inflammatory biomarkers and adverse outcomes.

Summary:
This manuscript describes the results of a study on “Intermittent preventive treatment with sulphadoxine-pyrimethamine but not dihydroartemisinin-piperaquine modulates the relationship between inflammatory markers and adverse pregnancy outcomes in Malawi”, by Kaylene et al.
They assessed the Relationship between inflammation and birth outcomes in women receiving IPTp using Sulfadoxine-pyrimethamine or dihydroartemisinin piperaquine.


The results indicate there that Elevated alpha-1-acid glycoprotein (AGP)AGP at enrollment was associated with adverse birth outcome, with similar associations observed across treatment arms, and
with low maternal weight gain in SP recipients only. At visit 3 there were few associations between inflammation and adverse outcomes. At delivery, women with elevated AGP receiving DP or DP+AZ had an increased risk of adverse birth outcomes, including LBW, PTB and foetal loss. The risk of an association between elevated AGP and adverse birth outcome was higher in those receiving DP or DP+AZ compared to those receiving SP. In contrast to AGP, no clear associations between CRP and adverse outcomes were observed. The authors concluded that SP modifies the relationship between inflammatory biomarkers and adverse outcomes.

General comments:
The results indicate there that Elevated alpha-1-acid glycoprotein (AGP)AGP at enrollment was associated with adverse birth outcome, with similar associations observed across treatment arms, and
with low maternal weight gain in SP recipients only. At visit 3 there were few associations between inflammation and adverse outcomes. At delivery, women with elevated AGP receiving DP or DP+AZ had an increased risk of adverse birth outcomes, including LBW, PTB and foetal loss. The risk of an association between elevated AGP and adverse birth outcome was higher in those receiving DP or DP+AZ compared to those receiving SP. In contrast to AGP, no clear associations between CRP and adverse outcomes were observed. The authors concluded that SP modifies the relationship between inflammatory biomarkers and adverse outcomes and suggested that their findings provide insights into the potential mechanisms by which SP may improve pregnancy outcomes.

Specific comments:
1) Abstract: Can the authors spelled out the terms LBW, PTB?
We have edited the abstract so it now reads as

Line 56 “including low birth weight (LBW), pre-term birth (PTB) and foetal loss”

2) Introduction:
Line 69: birthweigh is in single word, but in two words in other places (eg line 68). Please be consistent.

We have made it two words throughout the manuscript

Line 75: Can the authors add a reference at the end of the sentence?

We have added references to this sentence (Line 76)

3) Methods:

Line 119: can the authors add the reference of the approval letter for COMREC?

Line 121 Added to the methods- Ethics Committee (COMREC 01/19/2578).

Line 123. Sex is listed as variable that was recorded at birth. However, this variable is not presented anywhere in the results section. Is that variable less important to modify your conclusion?
We did not investigate the role of sex in the model, however we have included it because sex was used when calculating the z-score for foetal growth.

We have now included a reference for the calculation of outcome variables (including the z-scores) in the methods section (see lines 128 & 133).

Line 132: A reference is needed at the end of this sentence.

We have not included a reference as to our knowledge there is not a suitable one available, however we have added the product numbers used and there are details of the protocol described in the methods.

Line 136 “Human CRP and AGP concentrations in plasma were measured using DuoSet ELISA kits from R&D systems (DY1707 and DY3694).”

Line 133: The authors modified the protocols for use with 384 microwell plates. Can the authors explain the rational of that modification?

Thanks for this query, we adjusted the protocol to make it higher throughput, we have clarified this in the manuscript.

Line 136 “To facilitate the measurement of the high number of samples the protocols were modified for use with 384 microwell plates”

Line 162: The authors mentioned that Changes in inflammatory markers in 162 individual women were calculated by subtracting levels at enrollment from those at visit 3. Did the authors also assess the same changes from enrollment to delivery? If not, one would like to know that change.

We have added to figure 1 to show changes between enrolment and delivery, changes were similar in the three arms.

Line 227 ”Changes in inflammatory markers between enrolment and delivery were similar in all three arms (Figure 1E&F).”

Line 230 **“Figure 1: Distribution of AGP and CRP (A,B, G &H) and changes in AGP and CRP between enrolment and 24-32 weeks (C&D) and enrolment and delivery (E&F) by treatment arm.**

4. Results:

Table 1:
What is the rational of measuring the Mid upper arm circumference? This measure is not seen in any of the advance analysis. Same for the small for gestational age under the birth outcomes.

The reviewer is correct in that mid-upper arm circumference was not an outcome of interest, however we do include it in the model used in the paper. This information is stated in the methods and underneath the respective results tables. Mid-upper arm circumference was sometimes abbreviated to MUAC which may have caused confusion, we have replaced all references to MUAC with Mid-upper arm circumference it to make it clearer.

Notes underneath of table: Low birth weight (line 210) is in two words, but in one word in other places in the text.

Birthweight is now two words throughout the text

There is a typos gestational weight (line 215).
thank you- we have corrected this now to say ‘gestational’

Line 220 and line 221: There were elevation for both CRP and AGP at enrolment, followed by a decrease at visit 3 and then an increase at delivery. What are the hypotheses explaining these trends. In other words, why they both drop at visit 3 and increase at delivery?

We are not sure why there is a decrease between enrolment and visit 3 and increase at delivery but have edited the discussion to make it clearer how these changes fit within previous findings. Also please see comments to reviewer 1 point 4.

Line 380 “Concentrations of CRP and AGP fell between enrolment and visit 3, when there were few associations between inflammation and adverse outcomes and comparatively few women had inflammation, and then rose again at delivery. The mechanism underlying these changes are unclear, but similar declines in AGP between the end of the first and second trimester were reported from a study of pregnant women in Pakistan, India and Guatemala who did not receive IPTp (3) and an increase in CRP in the third trimester (compared to the first) has been previously seen in a pregnant women from Nepal (4).”

Line 231: Typos in “test was”

Thank you this has been corrected.

5. Discussion:
Line 405: … spectrum….. STIs. Reference should be added here.

We have added a more appropriate reference for SP and STI (5) (line 411) and have added a reference for broad action of AZ (6) (line 412).

Line 412 Typos in “ particpants”

This has been corrected to ‘participants.’

1.	Villar J, Cheikh Ismail L, Victora CG, Ohuma EO, Bertino E, Altman DG, et al. International standards for newborn weight, length, and head circumference by gestational age and sex: the Newborn Cross-Sectional Study of the INTERGROWTH-21st Project. Lancet. 2014;384(9946):857-68.

2.	Kim S, Naziripour A, Prabhala P, Horvath V, Junaid A, Breault DT, et al. Direct therapeutic effect of sulfadoxine-pyrimethamine on nutritional deficiency-induced enteric dysfunction in a human Intestine Chip. EBioMedicine. 2024;99:104921.

3.	Tang M, Weaver NE, Frank DN, Ir D, Robertson CE, Kemp JF, et al. Longitudinal Reduction in Diversity of Maternal Gut Microbiota During Pregnancy Is Observed in Multiple Low-Resource Settings: Results From the Women First Trial. Front Microbiol. 2022;13:823757.

4.	Sauder MW, Lee SE, Schulze KJ, Christian P, Wu LSF, Khatry SK, et al. Inflammation throughout pregnancy and fetal growth restriction in rural Nepal. Epidemiol Infect. 2019;147:e258.

5.	Chico RM, Chaponda EB, Ariti C, Chandramohan D. Sulfadoxine-Pyrimethamine Exhibits Dose-Response Protection Against Adverse Birth Outcomes Related to Malaria and Sexually Transmitted and Reproductive Tract Infections. Clin Infect Dis. 2017;64(8):1043-51.

6.	Neu HC. Clinical microbiology of azithromycin. Am J Med. 1991;91(3A):12S-8S.
